# Rapid-Onset Anti-Stress Effects of a Kappa-Opioid Receptor Antagonist, LY2795050, Against Immobility in an Open Space Swim Paradigm in Male and Female Mice

**DOI:** 10.3389/fphar.2021.775317

**Published:** 2021-11-22

**Authors:** Caroline Baynard, Thomas E. Prisinzano, Eduardo R. Butelman

**Affiliations:** ^1^ Laboratory on the Biology of Addictive Diseases, The Rockefeller University, New York, NY, United States; ^2^ Department of Pharmaceutical Sciences, College of Pharmacy, University of Kentucky, Lexington, KY, United States

**Keywords:** open space swim, kappa-opioid receptor, stress, acute stress disorder, dynorphin

## Abstract

The kappa-opioid receptor (KOR) / dynorphin system is implicated with behavioral and neurobiological effects of stress exposure (including heavy exposure to drugs of abuse) in translational animal models. Thus some KOR-antagonists can decrease the aversive, depressant-like and anxiety-like effects caused by stress exposure. The first generation of selective KOR-antagonists have slow onsets (hours) and extremely long durations of action (days-weeks), *in vivo*. A new generation of KOR antagonists with rapid onset and shorter duration of action can potentially decrease the effects of stress exposure in translational models, and may be of interest for medication development. This study examined the rapid onset anti-stress effects of one of the shorter acting novel KOR-antagonists (LY2795050, (3-chloro-4-(4-(((2S)-2-pyridin-3-ylpyrrolidin-1-yl)methyl) phenoxy)benzamide)) in a single-session open space swim (OSS) stress paradigm (15 min duration), in adult male and female C57BL/6 J mice. LY2795050 (0.32 mg/kg, i.p.) had rapid onset (within 15 min) and short duration (<3 h) of KOR-antagonist effects, based on its blockade of the locomotor depressant effects of the KOR-agonist U50,488 (10 mg/kg). LY2795050 (0.32 mg/kg), when administered only 1 min prior to the OSS stress paradigm, decreased immobility in males, but not females. With a slightly longer pretreatment time (15 min), this dose of LY2795050 decreased immobility in both males and females. A 10-fold smaller dose of LY2795050 (0.032 mg/kg) was inactive in the OSS, showing dose-dependence of this anti-stress effect. Overall, these studies show that a novel KOR-antagonist can produce very rapid onset anti-immobility effects in this model of acute stress exposure.

## Introduction

Acute stress exposure can cause behavioral and neurobiological changes (Reed et al., 2012; Carter et al., 2020; Varlinskaya et al., 2020; Becker et al., 2021), and can result in psychiatric sequeleae in humans, including acute stress disorder. The κ(kappa)-opioid receptors (KOR) and their endogenous ligands, the dynorphins, are one of the systems implicated in behavioral and neurobiological effects of stress exposure (Shirayama et al., 2004; Knoll and Carlezon, 2010; Reed et al., 2012; Chavkin, 2018). Thus, several studies have shown that the earliest known selective KOR-antagonists, including nor-binaltorphimine (nor-BNI), decreased immobility in the standard forced swim test (FST), as well as other behavioral consequences of stress exposure (Portoghese et al., 1987; Zhang et al., 2007; Carroll and Carlezon, 2013; Falcon et al., 2014). However, some of these early selective KOR-antagonists, such as nor-BNI, have slow onsets of action (e.g., several hours), and extremely long-lasting profiles *in vivo* (days-weeks, depending on the assay) (Horan et al., 1992; Broadbear et al., 1994; Butelman et al., 1998; Carroll and Carlezon, 2013). This may complicate their use in translational models of acute stress exposure and its consequences, and eventual medication development (Carroll and Carlezon, 2013; Page et al., 2019).

Several chemically unrelated shorter-acting KOR-antagonists have been developed more recently, and some have reached the clinical investigation stage (Aldrich et al., 2009; Chang et al., 2011; Rorick-Kehn et al., 2014; Huang et al., 2016; Valenza et al., 2017; Guerrero et al., 2019; Margolis et al., 2020; Valenza et al., 2020). We recently reported that one of the shorter acting KOR-antagonists, LY2795050, produced anti-immobility effects in the forced swim test (FST) in male, but not female, mice (Butelman et al., 2021). This is consistent with a sex difference observed with nor-BNI in the same assay (Laman-Maharg et al., 2018). Of translational relevance, ^11^C-LY2795050 has been used as a KOR positron emission tomography radiotracer in humans (Naganawa et al., 2015; de Laat et al., 2019), and is also structurally related to LY2456302 (aticaprant, CERC-501), which has reached the clinical investigation stage (Lowe et al., 2014; Reed et al., 2017). LY2795050 exhibits selectivity for KOR over mu-opioid and delta-opioid receptors (MOR and DOR) (Zheng et al., 2013). Thus, in cloned human receptors, the affinity (K_i_) of LY2795050 for KOR, MOR and DOR is 0.72, 25.8 and 153 nM respectively (Zheng et al., 2013). The antagonist potency of LY2795050 *in vitro* (K_b_) at KOR, MOR and DOR is 0.63, 6.8 and 83.3 nM respectively (Zheng et al., 2013).

The FST was originally developed as a screening test for anti-depressants, and its mechanistic underpinnings and interpretation have been extensively discussed (Porsolt et al., 1977; Porsolt et al.,1978; Chen et al., 2015; Molendijk and de Kloet, 2019; Armario, 2021). Recent reviews conclude that increased immobility in swim stress assays is reflective of a transition from active to passive coping, in the face of an inescapable stressor (Lanius et al., 2018; Molendijk and de Kloet, 2019; Armario, 2021). Several authors have also found that greater swim area allows more adaptive swimming behaviors for study (Jacobson M. L. et al., 2020; Armario, 2021). One modification of the FST assay is the open space swim (OSS) stress model, which due to its substantially greater available swim area, allows observation of more adaptive swim behaviors (Stone et al., 2008; Lin et al., 2011; Cates et al., 2013). In this study, we used a 1-day OSS procedure to examine in greater detail the rapid onset anti-stress effects of the KOR-antagonist LY2795050, as well as a follow-up to previously reported KOR-antagonist sex differences for the FST test (Laman-Maharg et al., 2018; Butelman et al., 2021).

## Methods

Subjects: Mouse studies were approved by the Rockefeller University Animal Care and Use Committee, consistent with the Guide for the Care and Use of Animals (National Academy Press; Washington, DC). Adult (9–10 weeks of age upon arrival to the facility), gonadally intact C57BL/6 J male and female mice were purchased from the Jackson Laboratory. The facility was approved by the American Association for Accreditation of Laboratory Animal Care (AAALAC). Mice were housed in one room at least 7 days prior to experiments. Mice were housed in same-sex groups (two to four per cage), in transparent plastic shoebox cages (19.6 cm wide X 30.9 cm long X 13.3 cm tall; Thoren Caging Systems, Hazleton, PA, United States). A n = 8 was the standard group size; this could vary slightly due to the availability of mice per cage. The cages had corncob bedding (“Bed-o’Cobs”, the Andersons, Maumee, OH, United States); shredded paper was added for nesting. The room was maintained at controlled temperature (≈20–22°C) and humidity, and had a 12:12 light:dark cycle (lights on at 0700), with overhead lighting. Experimental procedures occurred in the same room as housing. Procedures took place between 09:00 and 13:00 h.

### Apparatus

Locomotor activity: Locomotor activity was examined in mice placed individually in rectangular transparent plastic cages (19.7 cm width × 41.3 cm length × 20.3 cm height). Each bin had bedding identical to home cages and was placed in a photocell frame, which had an array of beams in perpendicular directions (SmartFrame; Kinder Scientific, Poway, CA). Beam breaks caused by the mouse were cumulated for analysis, on a computer interface.

Open Space Swim (OSS): This assay has been previously described (Stone et al., 2008) and was used with minor modifications, as a single session stress exposure. Mice were individually placed in a standard rectangular shoebox transparent plastic cage (19.7 cm width × 41.3 cm length × 20.3 cm height), with water at 31 ± 1°C, at a depth of approximately 13 cm. The swim surface area of the cage for OSS used here was therefore ≈814 cm^2^. For comparison, the standard FST test (not used here) provides an approximately 6-fold smaller swim surface area (typically 13 cm diameter; ≈133 cm^2^) (Porsolt et al., 1977; Castagne et al., 2011).

Sessions (15-min long) were recorded with digital videocameras, and analyzed by a trained observer, for “blind” scoring of duration of immobility. The camera was pointed horizontally, at the surface level of the water. This allowed visualization of movements above and below the surface.

### Procedures

Locomotor activity: One to three days prior to experiments, mice were habituated individually to locomotor cages, for a 60-min session. Consecutive experimental sessions in the same mice (90 min in duration) were separated by at least 72 h. Beam breaks were quantified as a measure of locomotor activity, over 15-min bins in the session.

Open Space Swim (OSS): A 1-day OSS procedure was carried out (Stone et al., 2008). Each mouse was used only once, in a single 15-min swim period. Immobility was defined as having only movements necessary to stay afloat, for ⩾2 s. After the test, the animal was returned to home cage. The water was changed after 8 mice had swum, or as needed to maintain water temperature. Immobility time was timed cumulatively in 1-min bins, by a trained observer who was “blinded” as to the treatment (i.e., vehicle or LY2795050). After training, intra-rater correlation for repeated scoring of 8 separate 1-min bins was high (Pearson *r* = 0.97; *p* < 0.0001), as was inter-rater correlation (Pearson *r* = 0.93; *p* = 0.001).

### Design

Locomotor activity: Locomotor experiments were performed to define basic KOR-antagonist effects of LY2795050, to be further examined in the OSS. Experiments were carried out with a within-subjects design, balanced for order. We determined the time course of the antagonist effects of LY2795050 (0.32 mg/kg, or vehicle) in preventing locomotor deficits caused by U50,488 (10 mg/kg), in males only. These dosing conditions were based on prior dose-effect studies, including determinations in both males and females (Butelman et al., 2019; Butelman et al., 2021). LY2795050 (0.32 mg/kg or vehicle) was therefore injected 15 min, 3 h, or 24 h prior to U50,488 (10 mg/kg). Mice were placed into locomotor cages immediately following U50,488 injection, for a 90-min session. In a second study, a 10-fold smaller dose of LY2795050 (0.032 mg/kg or vehicle) was injected as a 15 min pretreatment to U50,488 (10 mg/kg); this smaller LY2795050 was found to be inactive in other assays (Butelman et al., 2019; Butelman et al., 2021).

Open space swim (OSS): OSS studies were performed with a between-groups design; each mouse was used for a single session. In the first experiment, LY2795050 (0.32 mg/kg, or vehicle) was administered as a 1-min pretreatment to OSS in males and females. The second study examined the effects of the same dose of LY2795050 (0.32 mg/kg, or vehicle), but with a longer pretreatment time (15 min). Lastly, the effects of a 10-fold smaller dose of LY2795050 (0.032 mg/kg, or vehicle) was studied, with a 15-min pretreatment before OSS.

### Statistics

Graphpad Prism (version 9) software was used for statistical analyses. Data for locomotor activity and immobility were analyzed with repeated measures ANOVAs and Sidak’s or Tukey’s post-hoc tests (including planned comparisons), or with t-tests (*p* ≤ 0.05 level used as the criterion for significance). Mixed effects ANOVAs were used if there were missing data. Data for each 15-min OSS session were also analyzed as 1-min bins, in order to examine in greater detail the emergence of immobility over time. This was done with a non-linear regression with an exponential plateau growth curve (Graphpad Prism), with the following parameters: Y=Y_max_ -(Y_max_-Y_min_) * exp(-k*x), where Y_min_ and Y_max_ are the minimum and maximum value of the curve, respectively, “*k*” is the rate constant, and “*x*” is the time (1-min bins). Since these were 1-min bins measuring immobility, Y_min_ was constrained at 0 s, and the largest possible Y_max_ value was 60 s. A lack of overlap in the 95%CI for the maximum plateau value (Y_max_) between LY2795050 and vehicle treatment was used as an indicator of a significant difference.

### Drugs

As mentioned above, doses of the KOR-antagonist LY2795050 and the KOR-agonist U50,488 were based on recent reports (Butelman et al., 2019; Butelman et al., 2021). Thus, 0.32 mg/kg LY2795050 causes KOR-antagonist effects in different assays (including a locomotor assay) in both male and female C57BL/6 J mice (Butelman et al., 2021). By contrast, a 10-fold smaller LY2795050 dose (0.032 mg/kg) is inactive (Butelman et al., 2019; Butelman et al., 2021); see also (Placzek et al., 2018). U50,488 (10 mg/kg) produces robust decreases in locomotor activity in both male and female mice (Paris et al., 2011; Butelman et al., 2021). Therefore controls for these conditions were not carried out here, in order to limit the total number of mouse experiments. Non-radiolabeled LY2795050 (3-chloro-4-(4-(((2S)-2-pyridin-3-ylpyrrolidin-1-yl)methyl) phenoxy)benzamide)) (Naganawa et al., 2015; 2015b; Placzek et al., 2018) was purchased from BOC Sciences (Shirley, NY). LY2795050 was dissolved in 5% ethanol/10% Tween 80/85% sterile water, by volume. U50,488 was (Sigma-Aldrich) was dissolved in sterile water. LY2795050 and U50,488H were used within approximately 2 h of being in solution. Each experiment had its own appropriate vehicle condition. Mice were injected i.p. (volume of 10 ml/kg).

## Results

### Locomotor Activity

**FIGURE 1:**
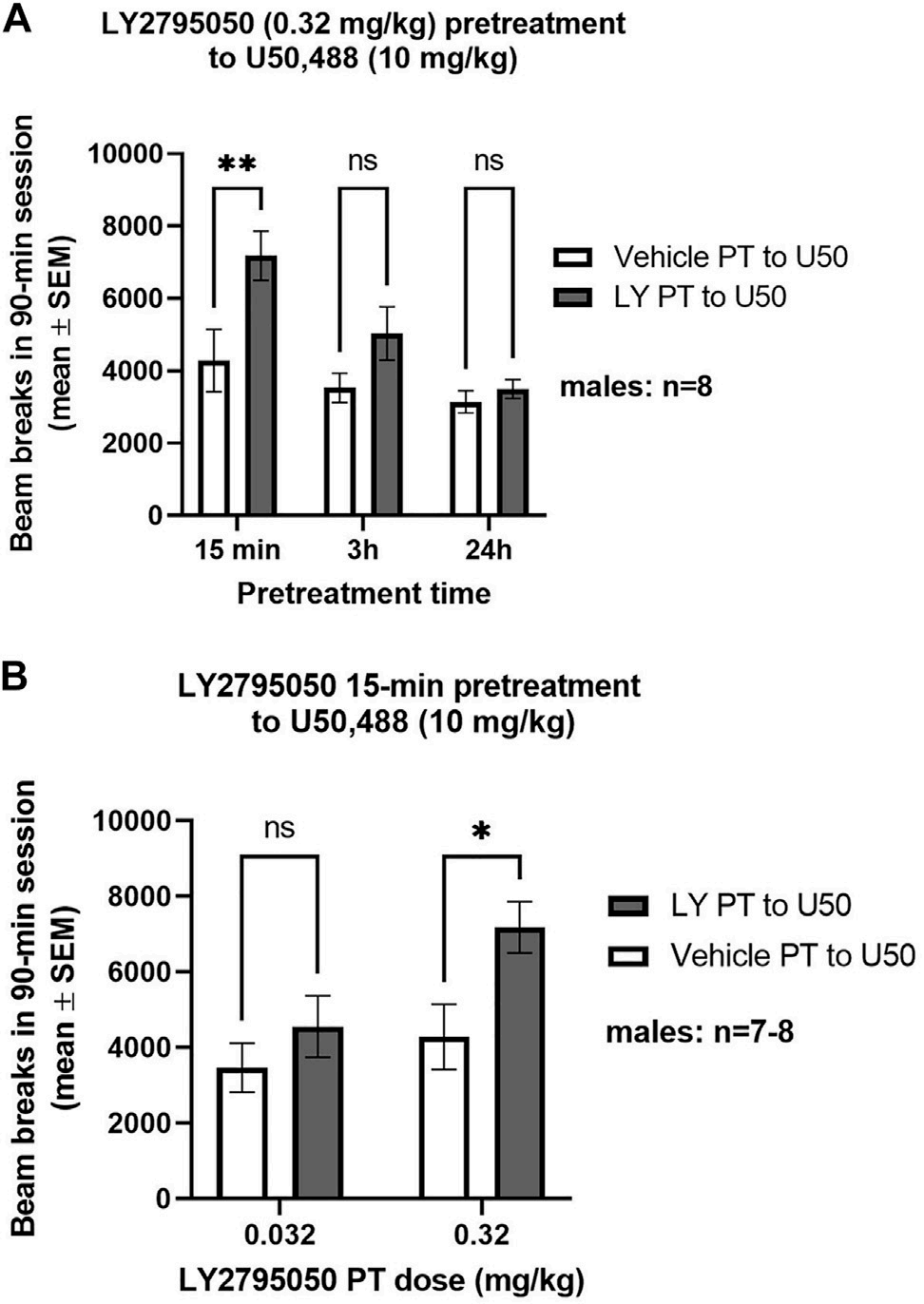
KOR-antagonist effects of LY2795050 pretreatment to U50,488 in the locomotor activity assay (in males). Total locomotor counts are shown for a 90-min session, starting immediately after injection of U50,488. **(A)**: Effect of LY2795050 (0.32 mg/kg or vehicle) given at different pretreatment times (15 min, 3 h, or 24 h) before U50,488 (10 mg/kg). **(B)**: Effect of different doses of LY2795050 (0.032 and 0.32 mg/kg, or vehicle) given 15 min before U50,488 (10 mg/kg).

LY2795050, at a dose that is devoid of direct locomotor effects (0.32 mg/kg) (Butelman et al., 2021), prevented the locomotor depressant effects of U50,488 (10 mg/kg) when given as a 15 min, but not 3 h or 24 h, pretreatment (only males studied; Figure 1A). A 2-way ANOVA [pretreatment (LY2795050 or vehicle) X pretreatment time] found a main effect of both pretreatment (F (1, 7) = 21.73; *p =* 0.0023) and pretreatment time [F (2, 14) = 20.85; *p* < 0.0001] but no interaction [F (2, 14) = 2.572; NS]. A Sidak’s multiple comparisons test determined that LY2795050 pretreatment significantly reduced U50,488-induced locomotor deficits compared to vehicle pretreatment, only at the shortest PT time (15 min).

The KOR-antagonist effect of LY2795050 in the locomotor assay was dose-dependent, as a 10-fold smaller dose (0.032 mg/kg), given as a 15 min pretreatment to U50,488, did not prevent U50,488-induced locomotor deficits (Figure 1B). A 2-way ANOVA (pretreatment X LY2795050 dose) revealed main effects of pretreatment [F (1, 13) = 7.351; *p* = 0.0178] and of LY2795050 dose [F (1, 13) = 4.79; *p* = 0.047]. There was no pretreatment *X* dose interaction [F (1, 13) = 1.509; NS]. Planned comparisons with Sidak’s tests revealed significant differences between the larger dose of LY2795050 (0.32 mg/kg) pretreatment and its vehicle condition, but not for the smaller dose (0.032 mg/kg) (Figure 1).

### Open Space Swim (OSS)

**FIGURE 2:**
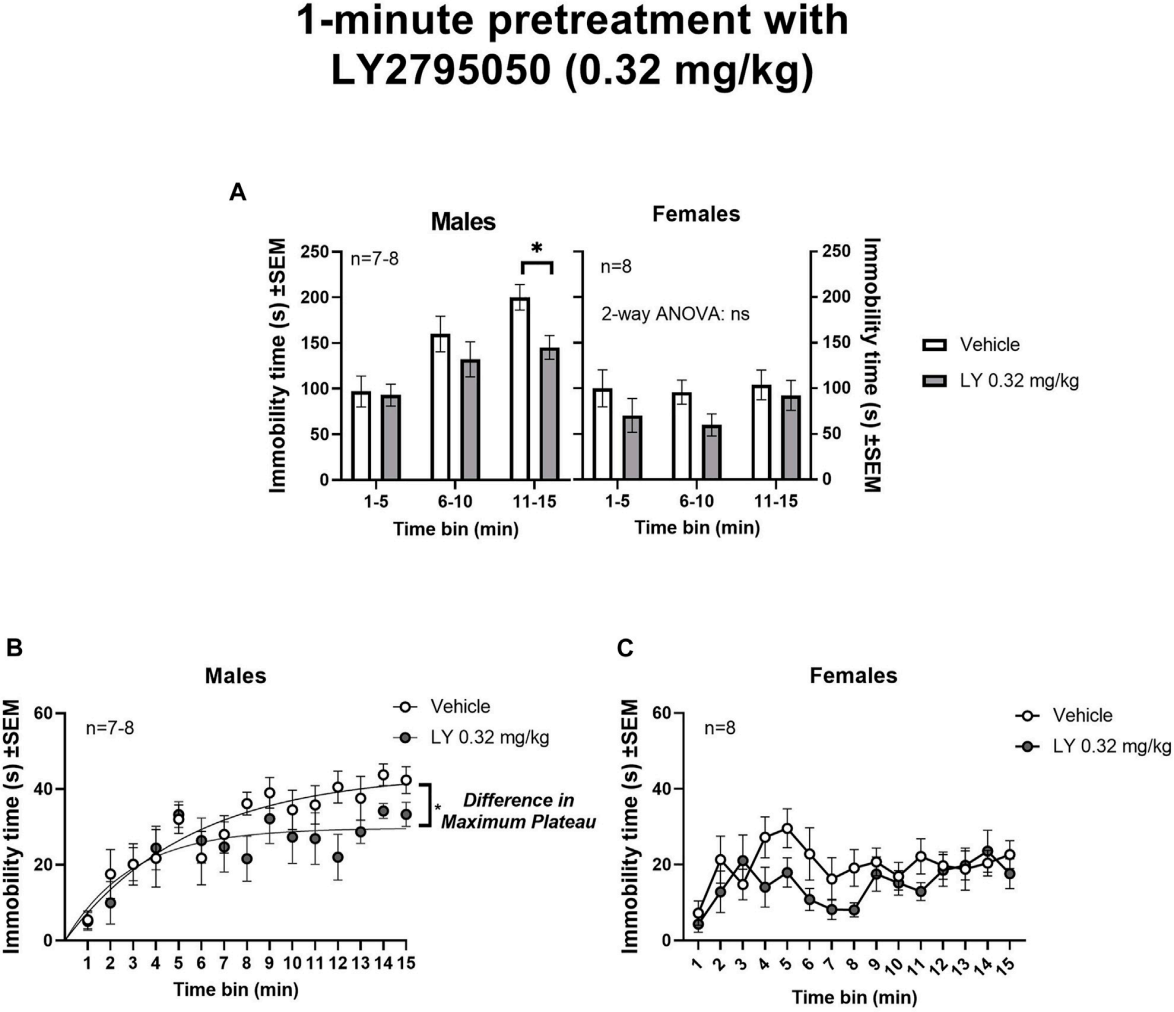
Effect of 1-min pretreatment of LY2795050 (0.32 mg/kg, or vehicle) on immobility in the open space swim (15-min duration). **(A)** shows data in three consecutive 5-min bins, analyzed by mixed model 2-way ANOVA, in males and females (the data were also analyzed in a 3-Way ANOVA; see Results). **(B,C)** show data re-plotted as 1-min bins, in males and females respectively. The regression lines in **(B)** (males) shows exponential plateau growth curves (see Methods). The fitted maximum plateau for immobility in males was significantly lower for LY2795050 versus vehicle (based on non-overlapping 95%CI). This regression could not be fit for panel C (females).

1-min pretreatment with LY2795050 (0.32 mg/kg): A 1-min pretreatment with LY2795050 (0.32 mg/kg) decreased immobility in the OSS session (15-min in duration) compared to vehicle, in males but not in females (Figure 2). In order to examine sex differences, data for 3 consecutive 5-min bins were analyzed with a 3-way mixed model ANOVA (treatment *X* sex *X* time bin, with repeated measures on the last variable) (Figure 2A). In this 3-way ANOVA, there was a main effect of treatment [F (1, 27) = 6.10; *p* = 0.02], of sex [F (1, 27) = 21.00; *p* < 0.0001] and of time bin [F (1.67,44.29) = 9.92; *p* = 0.0006]. There was also a significant interaction between sex and time bin [F (2, 53) = 6.20; *p* = 0.0038], but no other interaction was significant (not shown). Tukey’s post-hoc tests were used to examine sex differences. In the first two time bins (i.e., 1-5 and 6–10 min), there were no differences between males and females, either for the vehicle or LY2795050 conditions. However, in the third time bin (11–15 min), males had significantly greater immobility scores under the vehicle condition, compared to females (Tukey test; *p* = 0.0043). There were no sex differences in the LY2795050 condition.

In order to further explore the effects of LY2795050, we then analyzed the data separately for males and females. In males, a mixed-effects 2-way ANOVA of three consecutive 5-min time bins (treatment X time bin) detected a main effect of time bin [F (1.58, 19.76) = 14.50; *p =* 0.0003] but not of treatment [F (1, 13) = 3.87; ns] or their interaction [F (2,25) = 1.50; ns] (Figure 2A). A planned comparison with Sidak’s test showed a significant decrease in immobility only in the last bin (11–15 min), in the group pretreated with LY2795050 compared to vehicle. A non-linear regression was carried out to examine maximum immobility in greater detail across the OSS session (consecutive 1-min time bins; see Methods) (Figure 2B). This regression indicated a significantly lower maximum plateau value (Y_max_) for immobility in the LY2795050 versus the vehicle group, as denoted by a lack of overlap in 95%CI. Thus, the best fit value for maximum immobility after LY2795050 (29.7 s; 95%CI: 25.8–35.6) was lower than after vehicle (44.2 s; 95%CI: 37.4–58.3).

In females, no effect of LY2795050 was detected, in a similar 2-way ANOVA for 5-min bins, neither for treatment [F (1,14) = 2.45; ns], nor time bin [F (2,28) = 1.06; ns], or their interaction [F(2,28) = 0.4; ns] (Figure 2A). A non-linear fit could not be adequately achieved with the 1-min bin data in females (Figure 2C).

**FIGURE 3:**
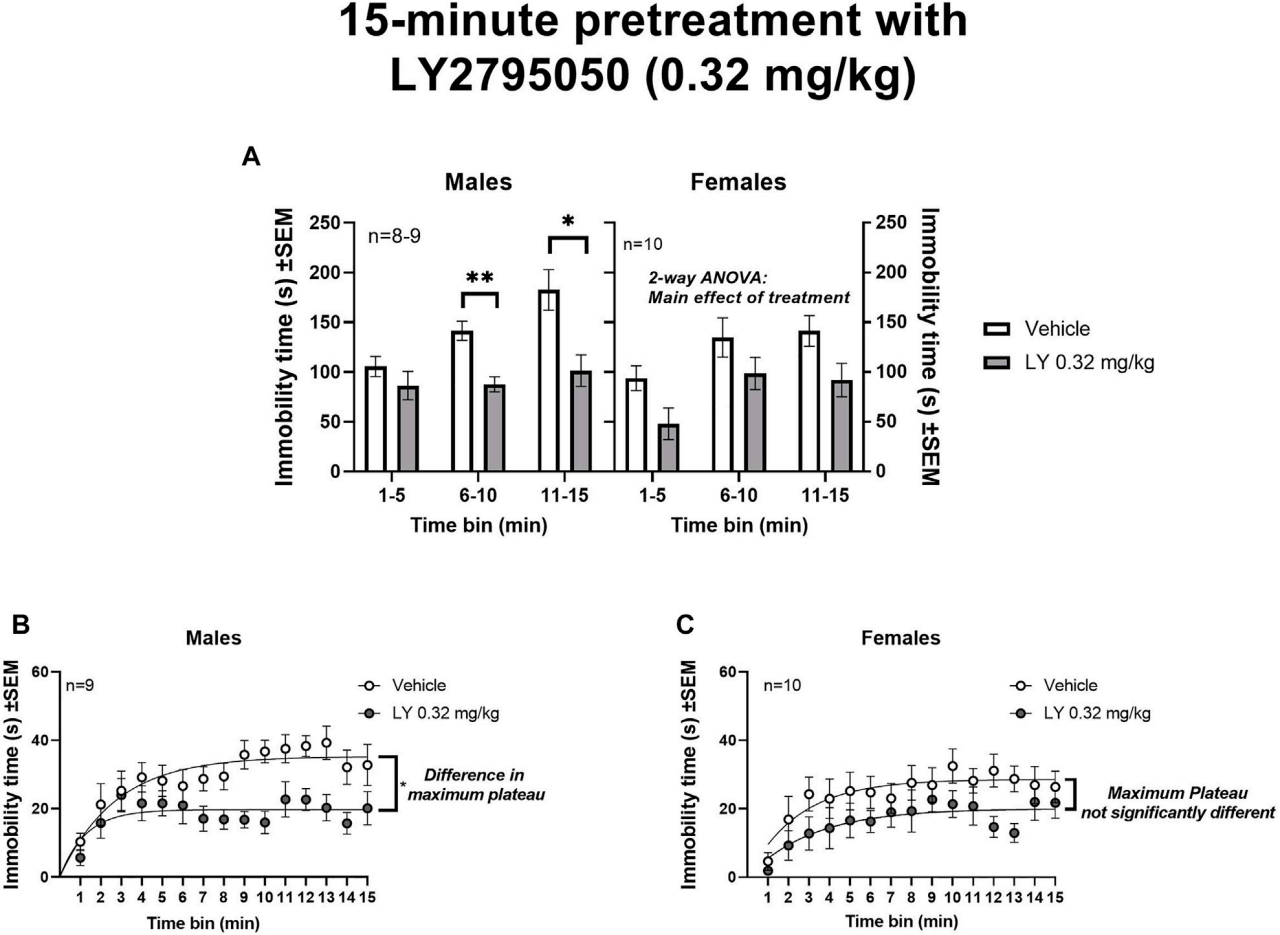
Effect of 15-min pretreatment of LY2795050 (0.32 mg/kg, or vehicle) on immobility in the open space swim (15-min duration). **(A)** shows data in three consecutive 5-min bins, analyzed by mixed model 2-way ANOVA, in males and females (the data were also analyzed in a 3-Way ANOVA; see Results). **(B,C)** show data re-plotted as 1-min bins, in males and females respectively. The regression lines in **(B)** (males) show exponential plateau growth curves; the fitted maximum plateau for immobility is significantly lower for LY2795050 versus vehicle. In **(C)** (females), the fitted maximum plateau is not significantly different for LY2795050 versus vehicle.

15-min pretreatment with LY2795050 (0.32 mg/kg): When administered with a slightly longer (15 min) pretreatment, LY2795050 decreased immobility in both males and females (Figure 2). As above, data for 3 consecutive 5-min bins were initially analyzed with a 3-way mixed model ANOVA (treatment X sex X time bin) (Figure 3A). In this 3-way ANOVA, there was a main effect of treatment [F (1, 33) = 26.46; *p* < 0.0001] and of time bin [F (1.73, 55.30) = 9.86; *p* = 0.0004], but no main effect of sex. There no significant interactions between these three variables (not shown). Likewise, there were no significant differences across sex, either in vehicle or LY2795050 conditions (Tukey’s tests).

We then further examined effects of LY2795050 in males and females separately, as above. In males, a 2-way mixed-effects ANOVA in three consecutive 5-min time bins (treatment X time bin) detected significant main effect of treatment [F (1, 44) = 21.97; *p* < 0.0001] and time bin [F (1.50, 33.07) = 5.87; *p =* 0.012], but no interaction [F (2, 44) = 2.65; ns] (Figure 3A). A planned comparison with Sidak’s test showed a significant decrease in immobility in the LY2795050-pretreated group compared to vehicle, in the last two time bins of the OSS (i.e., 6–10 min and 11–15 min bins). As above, a non-linear regression was used to examine immobility data in greater detail (1-min time bins) (Figure 3B, in males). This regression indicated a significantly lower maximum plateau value (Y_max_) for immobility in the LY2795050 versus the vehicle group. Thus, the best fit value for maximum immobility after LY2795050 (19.7 s; 95%CI: 17.5–22.1) was lower than after vehicle (35.3 s; 95%CI: 31.8–39.8).

In the females, a similar mixed-effects 2-way ANOVA over 5-min time bins (treatment X time bin) showed a main effect of treatment [F (1, 18) = 8.72; *p =* 0.0085] and time bin [F (1.76, 30.81) = 5.61; *p* = 0.011], but no interaction [F (2, 35) = 0.095; ns] (Figure 3A). Planned comparisons with Sidak’s test did not detect that any specific time bin was significantly different in LY2795050 versus vehicle conditions. A non-linear regression on the 1-min time bins in the females did not detect a difference in the maximum immobility in the LY2795050- versus the vehicle-treated mice (as shown by overlapping 95%CI) (Figure 3C). Thus, the best fit value for maximum immobility after LY2795050 (20.1 s; 95%CI: 16.8–25.6) was not different than after vehicle (28.6 s; 95%CI: 25.3–32.9).

15-min pretreatment with a 10-fold smaller dose of LY2795050 (0.032 mg/kg): At a 10-fold smaller dose (0.032 mg/kg) and with a 15-min pretreatment time, LY2795050 did not decrease immobility in the OSS, in either males or females. For example, in the last 5-min time bin of the session (i.e., 11–16 min), LY2795050- and vehicle-treated groups were not different, either in males or females (Table 1).

**TABLE 1:** Immobility in the last 5-min bin (i.e., 11–15 min) of the open space swim session after a 10-fold smaller dose of LY27950590 (0.032 mg/kg).

	0.032 mg/kg LY2795050	Vehicle	Unpaired *t*-test
	Mean (±95%CI)	Mean (±95%CI)	
Males (*n* = 8/group)	107.5 (52.7–162.4)	130.1 (89.4–170.8)	*t* (14) = 0.78; ns
Females (*n* = 8/group)	107.2 (56.2–158.1)	69.2 (33.4–105.0)	*t* (14) = 1.44; ns

## Discussion

This study shows that the short-acting KOR-antagonist LY2795050 can produce very rapid-onset anti-immobility effects in a model of acute stress exposure. The study also shows that apparent sex differences in the effects of KOR-antagonists may be due to the specific stress paradigm used (Laman-Maharg et al., 2018; Williams et al., 2018; Diester et al., 2019; Butelman et al., 2021).

Initial examinations in the locomotor activity assay demonstrated that LY2795050 (0.32 mg/kg) prevented U50,488-induced locomotor deficits when administered as a 15-min pretreatment, but was ineffective at longer pretreatments (3 and 24 h, determined only in males). This is consistent with the effectiveness of LY2795050 (0.32 mg/kg), previously determined in locomotor activity studies in both male and female mice (Butelman et al., 2021). The effectiveness of this dose of LY2795050, its rapid onset and relatively short duration of action are also consistent with prior data in rodents, in other assays (Placzek et al., 2018; Butelman et al., 2019). Other novel KOR antagonists, such as the congener LY2444296 (also known as FP3FBZ) (Melief et al., 2011; Valenza et al., 2017) have slightly longer durations of action than LY2795050 (Butelman et al., 2019). Duration of KOR antagonist action has been correlated to activation of c-Jun-N-terminal kinase-1 (JNK1) (Melief et al., 2011). The effects of LY2795050 on JNK1 activity have not been reported, to our knowledge.

We found for the first time that a very short (1 min) pretreatment with LY2795050 (0.32 mg/kg) was able to decrease immobility in the OSS assay (15-min duration), but only in male, but not female mice. Growth curve analysis over 1-min bins revealed that LY2795050 was able to decrease maximal immobility, which was observed typically in the last 5 min of the swim exposure.

Since KOR-antagonist effects of LY2795050 (0.32 mg/kg) were detected here in the locomotor assay with a 15-min pretreatment in males (and previously in females also) (Butelman et al., 2021), this pretreatment time was then studied in the OSS assay. With this slightly longer pretreatment, LY2795050 (0.32 mg/kg) caused a decrease in immobility in both male and female mice, when examined over the three consecutive 5-min bins of OSS stress exposure. Overall, these results indicate that short-acting KOR-antagonists such as LY2795050 can also produce anti-stress effects in females, depending on the assay, consistent with other reports (Williams et al., 2018; Jacobson et al., 2020c). Intriguingly, pretreatment with a different short-acting KOR antagonist prevented the effects of social defeat stress in both male and female California mice, whereas administration after the stress was ineffective (Williams et al., 2018). Potential sex differences in the behavioral effects of novel KOR-antagonists have been studied under a limited set of conditions (Williams et al., 2018; Jacobson et al., 2020a; Jacobson et al., 2020c; Butelman et al., 2021), but sexual dimorphism in the KOR/dynorphin system has been previously reported (Chartoff and Mavrikaki, 2015; Becker and Chartoff, 2019; Jacobson et al., 2020c).

The effects of LY2795050 in the OSS were dose-dependent, since a 10-fold smaller dose (0.032 mg/kg) did not affect immobility. This is consistent previous findings that this smaller dose of LY2795050 does not exhibit KOR-antagonist effects in other assays mice (Butelman et al., 2019; Butelman et al., 2021).

Limitations and methodological considerations: The largest dose of LY2795050 alone (0.32 mg/kg) does not have locomotor effects in either male or female C57BL/6 J mice (Butelman et al., 2021). Therefore the anti-immobility effects of LY2795050 in the OSS are not simply due to a confound with locomotor effects.

The time-dependent increase of immobility within a session of swim stress has been examined in the standard FST, previously (Castagne et al., 2009; Chen et al., 2015; Molendijk and de Kloet, 2019). In the standard FST in mice, immobility is typically reported for the last 4 min of a 6-min swim (Porsolt et al., 1977; Castagne et al., 2011). In order to examine the increase of immobility across the full 15-min OSS herein, we selected an exponential plateau growth curve, for nonlinear regression analysis. This regression model is appropriate for analysis of time series data where a maximum plateau is observed (as occurs in the OSS session). Using these regression analyses, we detected a LY2795050-induced decrease in the maximum plateau of immobility in males, but not in females. It cannot be excluded that a different regression model, or an increase in number of mice per group may have yielded slightly different results. Likewise, it cannot be excluded that there was a “floor” effect on immobility scores in the OSS in female mice.

In this study, male versus female differences in the effects of LY2795050 were examined in gonadally intact adult mice. Larger doses of LY2795050 (i.e., >0.32 mg/kg) were not studied, because they also exhibit MOR-antagonist effects (Butelman et al., 2019). Future studies could determine whether the subtle sex differences observed in the OSS were due to hormonal or organizational effects (Craft and Bernal, 2001; Land et al., 2009; Chartoff and Mavrikaki, 2015).

KOR and endogenous dynorphin function can be regulated by stress exposure in several brain areas and pathways (Shirayama et al., 2004; Carlezon et al., 2006; Rosa et al., 2018). Future studies could determine which brain pathway(s) mediate the rapid anti-stress effects of a systemically administered KOR-antagonist, such as LY2795050.

Conclusion: This is the first study to examine the effects of a KOR-antagonist in the open space swim stress (OSS) procedure. Very rapid onset anti-stress effects of LY2795050 were detected, more robustly in males than in females. This profile may be of potential interest in the modulation of the behavioral and neurobiological consequences of acute stress exposure, in translational models, and possibly for medication development.

## Data Availability

The raw data supporting the conclusion of this article will be made available by the authors, without undue reservation.

## Abbreviations

95%CI, 95%confidence interval; FST, Forced swim test; DOR, delta-opioid receptor; KOR, kappa-opioid receptor; MOR, mu-opioid receptor; LY, LY2795050 (short-acting KOR antagonist); NS, non-significant; OSS, Open space swim; PT, pretreatment.
